# Cosmid based mutagenesis causes genetic instability in *Streptomyces coelicolor*, as shown by targeting of the lipoprotein signal peptidase gene

**DOI:** 10.1038/srep29495

**Published:** 2016-07-12

**Authors:** John T. Munnoch, David A. Widdick, Govind Chandra, Iain C. Sutcliffe, Tracy Palmer, Matthew I. Hutchings

**Affiliations:** 1 School of Biological Sciences, University of East Anglia, Norwich Research Park, Norwich, UK; 2 Department of Molecular Microbiology, John Innes Centre, Norwich Research Park, Norwich, UK; 3 Department of Applied Sciences, Northumbria University, Newcastle, UK; 4 Division of Molecular Microbiology, College of Life Sciences, University of Dundee, Dundee, UK.

## Abstract

Bacterial lipoproteins are extracellular proteins tethered to cell membranes by covalently attached lipids. Deleting the lipoprotein signal peptidase (*lsp*) gene in *Streptomyces coelicolor* results in growth and developmental defects that cannot be restored by reintroducing *lsp*. This led us to hypothesise that *lsp* is essential and that the *lsp* mutant we isolated previously had acquired compensatory secondary mutations. Here we report resequencing of the genomes of wild-type M145 and the *cis*-complemented ∆*lsp* mutant (BJT1004) to map and identify these secondary mutations but we show that they do not increase the efficiency of disrupting *lsp* and are not *lsp* suppressors. We provide evidence that they are induced by introducing the cosmid St4A10∆*lsp*, as part of ReDirect PCR mutagenesis protocol, which transiently duplicates a number of important cell division genes. Disruption of *lsp* using a suicide vector (which does not result in gene duplication) still results in growth and developmental delays and we conclude that loss of Lsp function results in developmental defects due to the loss of all lipoproteins from the cell membrane. Significantly, our results also indicate the use of cosmid libraries for the genetic manipulation of bacteria can lead to phenotypes not necessarily linked to the gene(s) of interest.

Bacterial lipoproteins are essential for building and maintaining the cell envelope and they also provide a key interface with the external environment^1^,^2^,^3^. Most lipoprotein precursors are exported as unfolded polypeptides via the Sec (general secretory) pathway but others can be exported via the twin arginine transport (Tat) pathway, which is typically utilised for the transport of fully folded proteins^4^,^5^,^6^. The signal peptides of lipoproteins closely resemble other types of bacterial Sec and Tat signal peptide but they contain a characteristic lipobox motif, typically L_−3_-A/S_−2_-G/A_−1_-C_+1_, relative to the signal cleavage site, in which the cysteine residue is essential and invariant. The lipobox motif allows putative lipoproteins to be easily identified in bacterial genome sequences^3^,^7^. Following translocation, lipoprotein precursors are firstly modified by covalent attachment of a diacylglycerol molecule, derived from a membrane phospholipid, to the thiol of the conserved lipobox cysteine residue via a thioether linkage. This reaction is catalysed by an enzyme named Lgt (Lipoprotein diacylglycerol transferase) and results in a diacylated lipoprotein. Lsp (Lipoprotein signal peptidase) then cleaves the signal sequence immediately upstream of the lipidated cysteine to leave it at the +1 position. These early steps in lipoprotein biogenesis are highly conserved and unique to bacteria making them potential targets for antibacterial drug development^2^,^8^. In Gram-negative bacteria and Gram-positive Actinobacteria, lipoproteins can be further modified by addition of an amide-linked fatty acid to the amino group of the diacylated cysteine residue at the mature N-terminus. This final step is catalysed by the enzyme Lnt (Lipoprotein n-acyltransferase) and results in triacylated lipoproteins. In Gram-negative proteobacteria, Lnt modification is a pre-requisite for the recognition of lipoproteins by the Lol machinery, which transports lipoproteins to the outer membrane^2^,^9^ but its function in monoderm Gram-positive bacteria is not known. Members of the Gram-positive phyla Firmicutes and Mollicutes also N-acylate lipoproteins despite lacking Lnt homologues and *S. aureus* can diacylate or triacylate individual lipoproteins in an environmentally dependent manner^10^,^11^,^12^,^13^,^14^. These studies suggest that triacylation of lipoproteins in Gram-positive bacteria has an important role in their natural environment but is dispensable *in vitro.* Loss of Lnt activity in *Streptomyces* bacteria has no obvious effect on fitness or lipoprotein localisation *in vitro* but it does have a moderate effect on virulence in the plant pathogen *Streptomyces scabies*, supporting the idea that it has environmental importance^15^.

We previously characterised all four steps of the lipoprotein biogenesis pathway in *Streptomyces* spp. (Fig. 1)^5^,^15^, which is one of the best studied genera in the Gram-positive phylum Actinobacteria. Our key findings are (i) that Tat exports ~20% of lipoprotein precursors in streptomycetes; (ii) they N-acylate lipoproteins using two non-essential Lnt enzymes; (iii) *Streptomyces coelicolor* encodes two functional copies of Lgt which cannot be removed in the same strain; (iv) *lsp* mutants can be isolated at low frequencies but they acquire spontaneous secondary mutations which might be *lsp* suppressors. It was recently reported that Lgt is essential in *Mycobacterium tuberculosis*, which is also a member of the phylum Actinobacteria, and that *lgt* deletion in the fast-growing species *Mycobacterium smegmatis* is accompanied by spontaneous secondary mutations^16^. Natural product antibiotics that target the lipoprotein biogenesis pathway include globomycin, made by *Streptomyces globisporus*^2^ and antibiotic TA made by *Myxococcus xanthus*^1^,^16^. Both inhibit Lsp activity and are lethal to *Escherichia coli* but TA resistance arises through spontaneous *IS3* insertion into the *lpp* gene, which encodes an abundant lipoprotein that attaches the *E. coli* outer membrane to the peptidoglycan cell wall^16^,^17^. Over-expressing *lsp* also confers TA resistance in both *E. coli* and *M. xanthus*, and the latter encodes additional Lsp homologues within the TA biosynthetic gene cluster^17^.

Deletion of *S. coelicolor lsp* results in very small and flat colonies that are delayed in sporulation and these *lsp* mutants could not be fully complemented even by reintroducing the *lsp* gene to its native locus. Although both *cis* and *in trans* complementation restored lipoprotein biogenesis and sporulation it did not restore the wild-type growth rate^5^. There are two likely reasons for this: either *lsp* is essential and ∆*lsp* mutants acquire secondary suppressor mutations or the ReDirect PCR targeting method that we used to delete the *lsp* gene resulted in mutations independent of *lsp.* ReDirect is the name given to a protocol in which the Lambda Red system is used to PCR target genes of interest in a *Streptomyces* cosmid library in *E. coli* and the mutated cosmids are conjugated into *Streptomyces* species to select for mutants. Here we provide evidence to support the second hypothesis by demonstrating that introduction of a cosmid carrying an ~40 kb region of the *S. coelicolor* chromosome, including *lsp*, from *E. coli* to *S. coelicolor* leads to growth and developmental defects. We further show that *lsp* is non-essential but that deletion of the *lsp* gene results in small colonies that over-produce actinorhodin, as observed previously. These phenotypes must therefore be due to the loss of lipoproteins from the cytoplasmic membrane of *S. coelicolor*.

## Results

### Mapping secondary mutations in the *cis* complemented Δ*lsp* strain BJT1004

**Figure 2:**
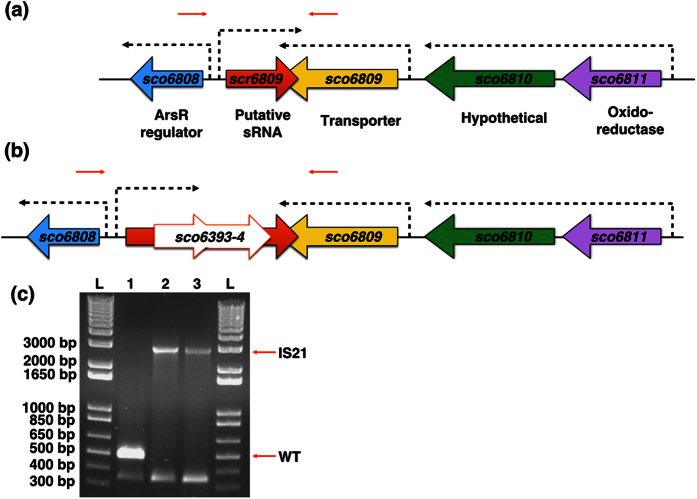
*IS21* insertion into *scr6809.* The *sco6811-08* region of the *S. coelicolor* M145 genome contains 4 genes (*sco6811* (Purple)*, sco6810* (green)*, sco6809* (yellow) and *sco6809* (blue) and a putative sRNA *scr6809* (large red) along with 3 putative promoters (broken arrows). Representations of the WT loci (**a**) and that sequenced from BJT1004 (**b**) indicate where an IS21 element (*sco6393* and sco6394) has inserted within *scr6809*. PCR verification of the IS21 insertion with primers JM0093 and JM0094 (small red arrows) was carried out (**c**) using M145 (lane 1), BJ1001 (lane 2) and BJT1004 (lane 3) genomic DNA. Lanes marked L contain the size ladders (Invitrogen 1 kb plus DNA ladder), lane 1 contains the PCR product using wild-type M145 DNA (514 bp), lane 2 contains the PCR product using ∆*lsp* strain BJT1001 DNA and lane 3 contains the PCR product using genomic DNA from the cis complemented ∆*lsp* strain BJT1004 (both 2884 bp.

**Figure 3:**
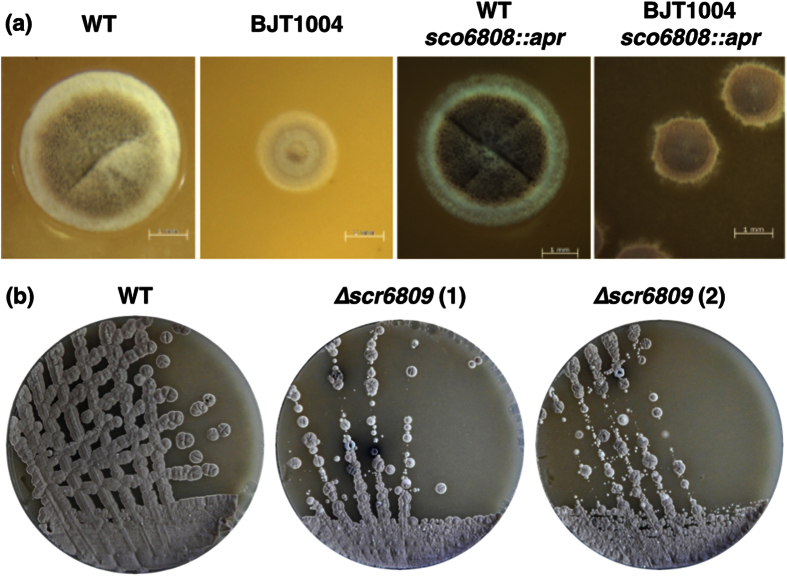
Analysis of the *IS21* disrupted genomic region in *S. coelicolor*. Colony morphology (**a**) shows that deletion of *sco6808* has no obvious effect on growth or development in wild-type M145 but does partially restore sporulation in BJT1004 (recovery of *scr6809*). Disruption of *scr6809* in M145 results in a range of pleiotropic morphological and developmental phenotypes (**b**).

We previously reported that the *S. coelicolor* ∆*lsp* mutant BJT1001 cannot be complemented even by restoring *lsp* to its native locus^5^. Since *cis* complementation should effectively restore the genome to wild-type this suggests that other spontaneous mutations have occurred during the deletion of *lsp*. To test this we resequenced the genomes of the isogenic parent strain *S. coelicolor* M145 and the *cis-*complemented ∆*lsp* strain BJT1004 using two independent companies and compared them with each other and with the published M145 sequence to identify mutations (Supplementary Table S1). Across all four sequences we identified a total of 51 single nucleotide polymorphisms (SNPs) as well as a chromosomal rearrangement in BJT1004 that is not present in the parent strain M145 (Fig. 2a,b and Supplementary Text S1 and S2). Of the 51 SNPs, 13 are unique to one of the BJT1004 sequences, with four residing inside coding sequences. Only one SNP occurs in both BJT1004 sequences and this is in the intergenic region between *sco5331* and *sco5332* and does not affect a coding sequence. In the single chromosomal rearrangement, the *IS21* insertion element (*sco6393*-*4*) has inserted into the intergenic region between *sco6808* and *sco6809* and this was confirmed by PCR (Fig. 2a–c). Although this could affect *sco6808* expression, deletion of *sco6808* has no effect on growth or development under standard laboratory conditions (Fig. 3a). This, and the intergenic position of *IS21* in BJT1004, led us to hypothesise that *IS21* might disrupt a non-coding RNA. Examination of RNA sequence data for *S. coelicolor* M145 confirmed the presence of a 189 nt transcript initiating 107 bp upstream of *sco6808* and reading into the last 82 nucleotides of the *sco6809* gene (data from GSM1121652 and GSM1121655 RNA sequencing; Supplementary Text S1 and 2; Fig. 2a,b). Following convention we named this putative small RNA *scr6809* for *S. coelicolor* RNA 6809. Deletion of *scr6809*, without disrupting the coding sequences of either *sco6808* or *sco6809*, resulted in pleiotropic effects, including colonies that look like wild-type and colonies defective in growth, aerial hyphae formation, sporulation or antibiotic overproducers. Restreaking ∆*scr6809* colonies with wild-type appearance gave rise to a range of colony morphologies, including growth and developmental defects (Fig. 3b). ∆*scr6809* colonies that were already defective in growth, development or antibiotic production maintained those phenotypes after restreaking. A previous report showed that a ∆*sco6808* mutant exhibits accelerated production of actinorhodin and undecylprodigiosin as well as precocious spore formation on R5 medium^18^. There was no observable difference between the wild-type and ∆*sco6808* strains under the growth conditions used here but disruption of *sco6808* in strain BJT1004 improved sporulation most likely because *scr6809* has been restored to its genome (Fig. 3a).

**Figure 4:**
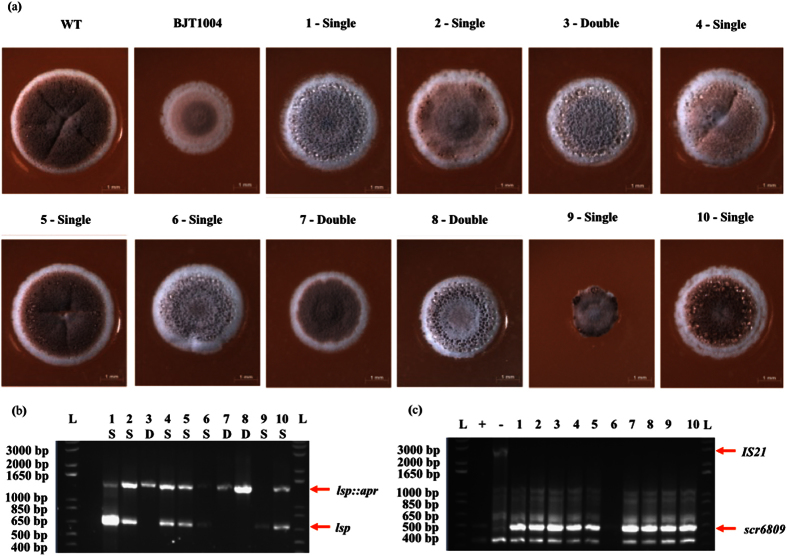
New *lsp* mutants generated using ReDirect do not contain the *IS21* mutation. Colony morphology of mutants *lsp::apr* 1–10 (corresponding to strains JTM008.01–JTM008.10), both single (n = 7, colonies 1–2, 4–7 and 9–10) and double cross-overs (n = 3, colonies: 3 and 7–8) show a range of phenotypes (**a**). PCR of the *lsp* loci (**b**) indicates colonies are either a single (wild-type and/or mutant band) or double (mutant band only) cross-overs (WT = 687 bp, mutant = 1447 bp). PCR of the *scr6809* loci (**c**) indicates that strains 1–10 have intact *scr6809* with no Insertion (WT = 514 bp, IS21 insertion = 2884 bp) using wild-type M145 and BJT1004 as controls (labeled “+” and “−” respectively).

To determine whether *IS21* disruption of *scr6809* is induced by deletion of *lsp*, we isolated ten more non-clonal *lsp* mutants. Of these 10 mutants, both single cross-over (n = 7) and double cross-over (n = 3) strains were isolated following the introduction of cosmid St4A10∆*lsp* into wild-type M145. Microscopy images of these strains showed a range of colony morphologies (Fig. 4a) and PCR results of the *lsp* loci confirm their genotypes, either single or double cross-over (Fig. 4b). These results suggest that secondary mutations are occurring in single cross-over strains containing intact *lsp* genes. The intergenic region between *sco6808* and *sco6809* was amplified to determine if these results were due to the disruption of *scr6809*. The size of the PCR products matched the predicted wild-type size for each strain and indicated that none of these *lsp* mutants contain an *IS21* insertion suggesting that the original observation is not specific to *lsp* mutants (Fig. 4c). Consistent with this conclusion, the frequency with which *lsp* mutants could be isolated was not increased in BJT1004 relative to the wild-type strain suggesting that none of the mapped mutations in BJT1004 are *lsp* specific suppressors. All attempts to over-express *scr6809* in *S. coelicolor* M145, *S. scabies* 87-22 and *S. venezualae* ATCC 10712 resulted in no observable phenotype but as the same vectors failed to complement the ∆*scr6809* strain, this suggests that functional *scr6809* was not expressed from vector pJM017. Cumulatively these results show that deletion of *lsp* is not solely responsible for the secondary mutations and this led us to hypothesise that these mutations accumulate as a result of duplicating cell division genes when we introduced cosmid St4A10∆*lsp.* These results also suggest a role for *scr6809* in development, but this was not pursued in this work.

### Introduction of wild-type St4A10 results in a pleiotropic phenotype

**Figure 5:**
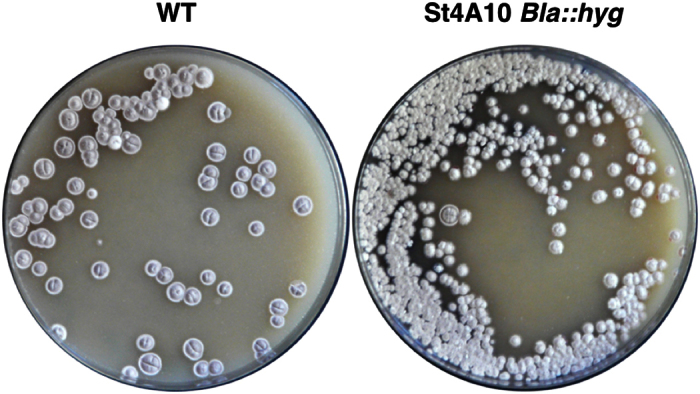
Introduction of wild-type St4A10 causes a pleiotropic phenotype. Conjugation of M145 with St4A10 *bla::hyg* results in non-wildtype phenotypes similar to those observed in the St4A10 *lsp::apr* single cross-overs.

The ReDirect PCR-targeting method^19^,^20^ used to delete the *lsp* gene utilized cosmid St4A10, which contains a ~40 kb region of the *S. coelicolor* genome spanning genes *sco2069*–*2104* (Supplementary Table S2). Conjugation of St4A10∆*lsp* into *S. coelicolor* transiently duplicates all the genes on that cosmid (except *lsp*) and because this region includes cell division genes (*ftsZ*, *ftsQ*, *ftsW*, *ftsI* and *ftsL*) and essential cell wall synthesis genes (*murG*, *murD*, *murX*, *murF* and *murE*) we reasoned that transient over-expression of these genes, rather than deletion of *lsp*, is responsible for at least some of the spontaneous secondary mutations and the resulting pleiotropic phenotype. To test this idea we introduced an origin of transfer into the wild-type St4A10 cosmid backbone and then conjugated this cosmid into wild-type *S. coelicolor* M145. We used growth in the presence of kanamycin to select for single cross-over events where the whole cosmid is integrated into the chromosome, thus duplicating the *S. coelicolor* genes on St4A10. Analysis of these single cross-over strains revealed them to be genetically unstable, with many initially appearing similar to the observed ∆*lsp* phenotype, i.e. forming small colonies that are delayed in sporulation (Fig. 5). However, they do not over-produce the blue antibiotic actinorhodin which was an obvious characteristic of *S. coelicolor* ∆*lsp.* In addition, colonies arising from the M145::St4A10 strain acquired more significant developmental issues upon prolonged maintenance and restreaking onto MS agar containing kanamycin (not shown). This suggests that this strain accumulates spontaneous secondary mutations as a direct result of carrying two copies of the genes on cosmid St4A10 and also supports our hypothesis that the observed ∆*lsp* phenotype is at least in part due to duplication of the genes on cosmid St4A10. This is consistent with the fact that complementation of ∆*lsp* restored lipoprotein biogenesis and all detectable lipoproteins to the cell membrane but did not restore wild-type colony morphology^5^.

### Targeted deletion of *lsp* results in a small colony phenotype

**Figure 6:**
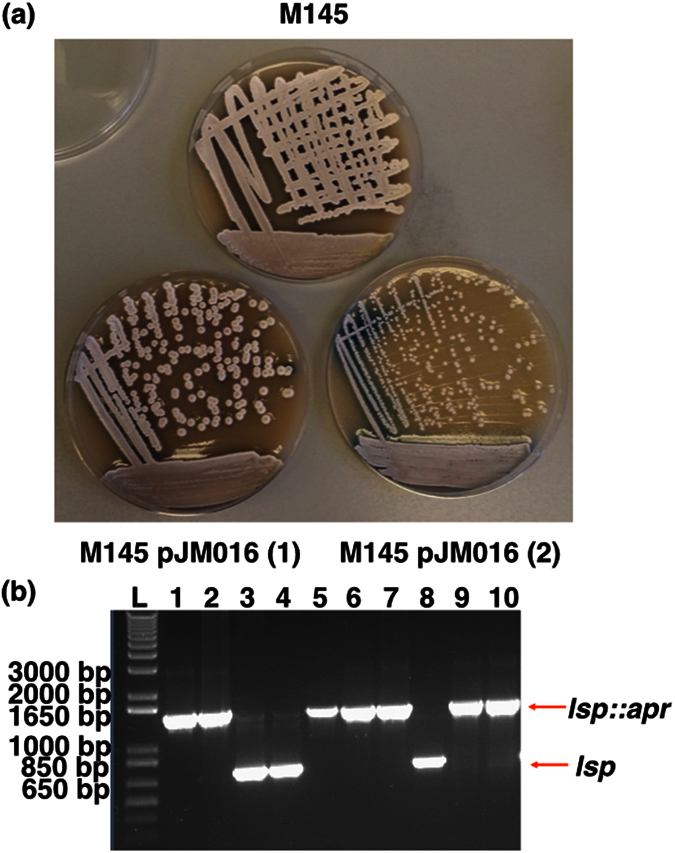
Targeted disruption of *lsp* using a suicide vector results in a small colony phenotype that over-produces actinorhodin. To test how much of the BJT1001 phenotype is due to loss of *lsp* we disrupted the *lsp* gene using a suicide vector which does not affect or duplicate any other target genes. Plate images (**a**) show two distinct phenotypes following insertion of the suicide vector (pJM016) into M145, either a wildtype appearance (M145 pJM016 (1), n = 3 corresponding to JTM018.03-04 and 08) or a small colony phenotype over producing actinorhodin (M145 pJM016 (2), n = 7, corresponding to strains JTM018.01-2, 05-07 and 09-10) similar to our original observation of the *lsp* phenotype^5^, alongside *lsp* loci PCR results (**b**). All strains with intact *lsp* show a wild-type phenotype while those with disrupted *lsp* have the reported *lsp* phenotype.

To test how much deletion of *lsp* gene alone contributed to the phenotype of BJT1001 (the ∆*lsp* strain generated using ReDirect) we undertook a targeted disruption of *lsp* in wild-type *S. coelicolor* M145 using a suicide vector, which does not duplicate or affect any other coding sequences. The *lsp* suicide vector, pJM016 (Table 1), was introduced into wild-type *S. coelicolor* by conjugation and ex-conjugants were selected by growing on MS agar plates containing apramycin. Following introduction of the pJM016, two colony types were observed (Fig. 6), one with wild-type appearance and the other with small colonies that over-produce actinorhodin, reminiscent of the *lsp* mutant BJT1001. PCR testing of the genomic DNA of both morphotypes revealed that those with the wild-type colony morphology are indeed wild-type strains with a fully functioning *lsp* gene whereas those with a small colony phenotype that over-produce actinorhodin have disruptions in *lsp* caused by pJM016. PCR amplification and sequencing of the loci in the small colony variants revealed an interesting and unexpected recombination event had occurred. The vector and almost all of the *lsp* gene have been removed such that all that remains is the apramycin resistance cassette (Supplementary Text S4 and S5). These data confirm that *lsp* is not essential in *S. coelicolor* and that loss of Lsp function (and the resulting loss of lipoproteins from the cell membrane) results in a growth and developmental defect and overproduction of the blue antibiotic actinorhodin, as observed previously^5^.

## Discussion

The results presented here show that the pleiotropic nature of *S. coelicolor* ∆*lsp* strain BJT1001 resulted from the introduction of cosmid St4A10∆*lsp* and this was most likely caused by over-expression of the cell division and cell wall biosynthesis genes carried on that cosmid (Supplementary Table S2). We have further shown that the resulting secondary mutations do not make it easier to delete *lsp* suggesting they are not *lsp-*specific suppressors. Genetic manipulation has always been challenging in *Streptomyces* bacteria and the ReDirect PCR targeting method 13 years ago was a significant development but our work is a cautionary tale to others to consider the effects of using large insert cosmid libraries in the genetic manipulation of bacteria. Fortunately, recent advances in CRISPR/Cas9 editing of *Streptomyces* genomes^21^ negate the need for a cosmid library and techniques such as this will further accelerate research into the basic biology of *Streptomyces* and other filamentous actinomycetes. This is vital because the secondary metabolites derived from these bacteria are an under utilised reservoir from which new anti-infectives and other drugs can and must be developed. Moreover, our identification of a new small RNA *scr6809* and the demonstration that its deletion results in a range of growth and developmental defects add to the growing appreciation of the significance of small RNAs in streptomycetes^22^,^23^,^24^.

## Materials and Methods

### Bacterial strains and culture conditions

All primers, plasmids and strains used are listed in Table 1. Strains were routinely grown as previously described^5^ following the recipes of Kieser *et al.*^25^. *E. coli* was grown in LB or LB –NaCl for Hygromycin selection and *S. coelicolor* M145 and its derivatives were grown on Soya Flour Mannitol (SFM) medium to study growth and development or LB culture for genomic isolations.

### Gene deletions and complementation

Gene deletions were carried out following the ReDirect method of PCR-targeting^26^ as previously described Hutchings *et al.*^27^. Disruption of *lsp (sco2074::apr)* on cosmid St4A10 (pJM013, St4A10∆*lsp*) using the pIJ773 apramycin disruption-cassette and *sco6808 (sco6808::*a*pr)* and *scr6809 (scr6809::apr)* on cosmid St1A2 (pJM010-St1A2∆*sco6808* and pJM012-St1A2∆*sco6808* respectively) using primers JM0101-2, JM0083-84 and JM0091-2 respectively were confirmed by PCR using primers JM0150-1, JM0085-6 and JM0093-4 respectively. Introduction of the wild-type cosmid St4A10 was facilitated by introducing an *oriT* by disruption and replacement of the Supercos-1 backbone *bla* resistance gene (pJM014–St4A10*bla::hyg)* using primers JM0095-6 and the hygromycin disruption cassette from pIJ10701, confirmed using primers JM0099-100. The *lsp* suicide vector pJM016 was produced by introducing a 411 bp fragment of the *lsp* gene with an N-terminal *Bam*HI site, amplified with primers JM0117-8 and cloned into pGEM T-Eazy. The *Bam*HI site was then used to subclone the *Bam*HI fragment from a pIJ773 digest, containing an *apr* disruption cassette. An overexpression construct, pJM017 was synthesised by Genscript to include the pMC500 MCS and terminators^28^ with *scr6809* (sequence included in Supplementary Text S5). All constructs were subsequently conjugated into *S. coelicolor* following the method described by Gust *et al.*^26^.

### Genomic DNA isolation

Genomic DNA was isolated from M145 and BJT1004 following the Pospiech and Neumann (1995) salting out method as described by Keiser *et al.* (2000). Mycelium from a 30 ml culture was resuspended in 5 ml SET buffer containing 1 mg/ml lysozyme and incubated at 37 °C 30–60 min. To this lysate, 140 μl of proteinase K solution (20 mg ml^−1^) was added, mixed, then 600 μl of 10% SDS added, mixed and incubated at 55 °C for 2 h, with occasional mixing throughout. After this incubation 2 ml of 5 M NaCl was added, mixed and left to cool to 37 °C before adding 5 ml chloroform, mixed at 20 °C for 30 min. Samples were centrifuged at 4500 × g for 15 min at 20 °C. The supernatant was removed to a fresh tube and DNA precipitated by adding 0.6 volumes of 100% isopropanol. Tubes were mixed by inversion and after at least 3 min DNA spooled out using a sterile Pasteur pipette. The DNA was rinced in 70% ethanol, air dried and dissolved in 1–2 ml TE buffer (10 mM Tris-HCl pH 7.8, 1 mM EDTA) at 55 °C.

### Genome resequencing and secondary mutation identification

The isolated DNA from our wild-type *S. coelicolor* M145 parent strain and BJT1004 were sent to both GATC Biotech and The Genome Analysis Centre (TGAC) for 35 bp paired end HiSeq Illumina sequencing. Assembly mapping and SNP identification was carried out with MIRA (Chevreux *et al.*, 2004) using the reference genome NC_003888 (Bentley *et al.*^29^) as a scaffold for mapping each of the resequenced genomes. Putative SNPs were detected in each sample independently reporting the SNP position, the nucleotide change, the number of reads that sequence the region, those containing wild-type or mutated nucleotides and a percentage change. Each set of results was then compared by eye to determine the likely hood that a SNP was real by number of reads containing the mutation and its presence in each sample. Larger mutations (rearrangements) were identified in the same fashion.

### Microscopy

Brightfield images were acquired using a Zeiss M2 Bio Quad SV11 stereomicroscope. Samples were illuminated from above using a halogen lamp images captured with an AxioCam HRc CCD camera. The AxioVision software (Carl Zeiss, Welwyn Garden City, UK) was used for image capture and processing.

## Additional Information

**How to cite this article**: Munnoch, J. T. *et al.* Cosmid based mutagenesis causes genetic instability in *Streptomyces coelicolor*, as shown by targeting of the lipoprotein signal peptidase gene. *Sci. Rep.* **6**, 29495; doi: 10.1038/srep29495 (2016).

## Supplementary Material

Supplementary Information

## Figures and Tables

**Figure 1:**
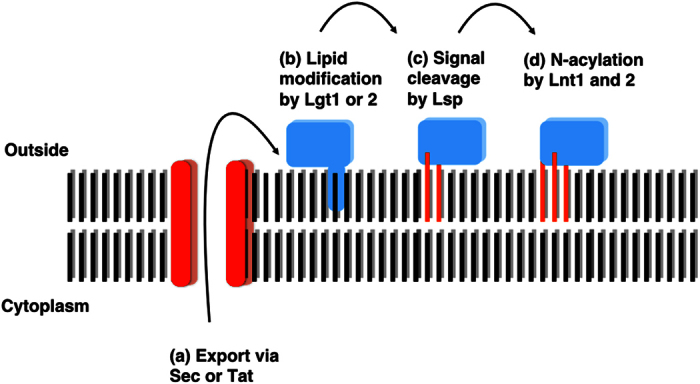
Lipoprotein biogenesis in *Streptomyces coelicolor.* Approximately 80% of precursor lipoproteins in *S. coelicolor* are translocated via the general secretory (Sec) pathway with around 20% being translocated by the twin arginine transport (Tat) pathway (**a**). Following translocation across the cytoplasmic membrane they are diacylated on the thiol of the lipobox (+1) cysteine residue by Lgt1 or Lgt (**b**) and then the signal sequence is cleaved by Lsp immediately upstream of that modified cysteine (**c**). Lnt1 then adds a third acyl chain to the amino group on the +1 cysteine to produce a triacylated lipoprotein (**d**). Lnt2 is not essential for triacylation *in vitro* but appears to increase its efficiency. The function of the N-acyl modification is not yet known.

**Table 1:** Strains, plasmids and primers.

Strain	Genotype/description	Plasmid (held)	Resistance	Source
*E. coli*				
TOP10	F– *mcrA* Δ(*mrr-hsdRMS-mcrBC*) Φ80*lacZ*ΔM15 Δ*lacX74 recA1 araD139* Δ(ara leu) 7697 *galU galK rpsL* (StrR) *endA1 nupG*	—	—	Invitrogen
BW25113	F-, DE(*araD-araB*)567, *lacZ4787*(del)::*rrnB-3*, LAM-, *rph-1*, DE(*rhaD-rhaB*)568, *hsdR514*	pIJ790	CmR	Datsenko & Wanner^30^
ET12567	dam- dcm- hsdM-	pUZ8002	CmR/TetR	MacNeil *et al.*^31^
*Streptomyces*	Genotype/description	Plasmid (used)	Resistance	Source
M145	*S. coelicolor* wild type strain, SCP1-, SCP2-	—	—	Hopwood *et al.*^32^
BJT1000	M145 *lsp::apr*	—	AprR	Thompson *et al.*^5^
BJT1001	M145 *lspFLP*	—	—	Thompson *et al.*^5^
BJT1004	BJT1000 + Sco *lsp* cis	—	—	Thompson *et al.*^5^
JTM005	M145 *sco6808::apr*	pJM010	AprR	This work
JTM007	M145 *scr6809::apr*	pJM012	AprR	This work
JTM008	M145 *lsp::apr*	pJM013	AprR	This work
JTM009	M145 St4A10 *bla::hyg*	pJM014	KanR/HygR	This work
JTM012	BJT1004 *sco6808::apr*	pJM010	AprR	This work
JTM015	BJT1004 *lsp::apr*	pJM013	AprR	This work
JTM018	M145 *lsp* suicide vector	pJM016	AmpR/AprR	This work
Plasmids	Genotype/description		Resistance	Source
pIJ773	*aac(3)IV oriT* (contains apramycin (apr) resistance cassette)		AprR	Gust *et al.*^26^
pIJ10700	contains hygromycin resistance cassette, FRT *oriT-hyg* FRT MkII		HygR	Gust *et al.*^26^
pIJ790	*araC-Parab, Υ, β, exo, cat, repA1001ts, oriR101*		CmR	Gust *et al.*^26^
pUZ8002	RK2 derivative with a mutation in *oriT*			Kieser *et al.*^25^
pMS82	*ori,* pUC18*, hyg, oriT, RK2, int ΦBT1*		HygR	Gregory *et al.*, 2003
pIJ10257	oriT, ΦBT1 attB-int, Hygr, ermEp*, pMS81 backbone		HygR	Hong *et al.*, 2005
pGEM-T-Eazy	*bla, lacZα*		AmpR	Promega
St1A2	Supercos-1-cosmid with (39829 bp) fragment containing (*sco6808* and *scr6809*)		KanR/AmpR	Redenbach *et al.*^20^
St4A10	Supercos-1-cosmid with (43147 bp) fragment containing (*sco2074-lsp*)		KanR/AmpR	Redenbach *et al.*^20^
pJM010	St1A2 containing *sco6808::apr oriT* (St1A2∆*sco6808*)		KanR/AmpR/AprR	This work
pJM012	St1A2 containing *scr6809::apr oriT* (St1A2∆*scr6809*)		KanR/AmpR/AprR	This work
pJM013	St4A10 containing sco2074::apr oriT (St4A10∆*lsp*)		KanR/AmpR/AprR	This work
pJM014	St4A10 containing *bla::hyg oriT* (St1A2*bla::hyg*)		KanR/HygR	This work
pJM015	pMS82 containing full length *sco2074* and promoter (300 bp of upstream DNA)		HygR	This work
pJM016	*lsp* suicide vector, pGEM-T-Eazy, 411 bp fragment of the *lsp* gene with a BamHI site. The *aac(3)IV* containing BamHI fragment from a pIJ773 was sub cloned in.		AmpR/AprR	This work
pJM017	pMS82, KpnI/HindIII insert containing, pMC500 MCS and terminators with *scr6809 (see Text S3)*		HygR	This work
Primer	Sequence	Description		Source
JM0083	GTCTATGGTTGACGGGTGACTGTCATAGATCTGCAGATGATTCCGGGGATCCGTCGACC	*sco6808* forward disruption primer (ReDirect)		This work
JM0084	GTCATCTTCCGAACGGAGATGGAGGGAGATCCGGAATCATGTAGGCTGGAGCTGCTTC	*sco6808* reverse disruption primer (ReDirect)		This work
JM0085	CGGAGGCCGCCTGTCCTAGC	*sco6808* forward test primer		This work
JM0086	AACGCGCACTCGCTGCGGTC	*sco6808* reverse test primer		This work
JM0091	TCCGACATCTGCAGATCTATGACAGTCACCCGTCAACCAATTCCGGGGATCCGTCGACC	*scr6809* forward disruption primer (ReDirect)		This work
JM0092	TGGTACACGGCACCGACTCCGGCTGCCAGAAAGCCATAGTGTAGGCTGGAGCTGCTTC	*scr6809* reverse disruption primer (ReDirect)		This work
JM0093	CAGACGCAGGCCTCGCCATC	*scr6809* forward test primer		This work
JM0094	CCCATCGCTACGGCCGCCT	*scr6809* reverse test primer		This work
JM0093	AATCAATCTAAAGTATATATGAGTAAACTTGGTCTGACAGTCAGGCGCCGGGGGCGGTG	*bla (bla::hyg)* gene forward disruption primer (ReDirect) for supercos-1		This work
JM0096	CCCTGATAAATGCTTCAATAATATTGAAAAAGGAAGAGTAAGTTCCCGCCAGCCTCGCA	*bla (bla::hyg)* gene forward disruption primer (ReDirect) for supercos-1		This work
JM0097	AAGCAGCAGATTACGCGCAG	*bla (bla::hyg)* gene forward test primer (ReDirect) for supercos-1		This work
JM0098	GTGCGCGGAACCCCTATTTG	*bla (bla::hyg)* gene forward test primer (ReDirect) for supercos-1		This work
JM0099	TCGTGCTCAGTCAAGGACCTAGGCTGAGGGACTCACGTGATTCCGGGGATCCGTCGACC	*lsp* (*sco2074*) forward disruption primer (ReDirect)		This work
JM0100	GACAACCAGTCCCTGTGGACAGCCGGACCGGAGGGGTCATGTAGGCTGGAGCTGCTTC	*lsp* (*sco2074*) reverse disruption primer (ReDirect)		This work
JM0113	GCAACAGTGCCGTTGATCGTGCTATG	pMS82 cloning forward test primer		This work
JM0114	GCCAGTGGTATTTATGTCAACACCGCC	pMS82 cloning reverse test primer		This work
JM0115	GGATCCCTGTTCGCGGTCGCCCTGTTCGCGTACCT	Forward primer amplifies a 411 bp fragment of the *lsp* gene, adding a bamHI site upstream.		This work
JM0116	GATGCCGCCGCACACGATCGCCGAGTCGG	Reverse primer amplifies a 411 bp fragment of the *lsp* gene		This work
JM0150	TCGTGCTCAGTCAAGGACCT	Sco Lsp Test For		Thompson *et al.*^5^
JM0151	GACAACCAGTCCCTGTGGAC	Sco Lsp Test Rev		Thompson *et al.*^5^
JM0154	AAGCTTCGACGAGGCGGACACAGGCAG	*lsp* (*sco2074*) comp (pMS82) for HindII		This work
JM0155	GGTACCTCAGTCCTTGTGGACGGTCCCGTC	*lsp* (*sco2074*) comp (pMS82) Rev KpnI		This work
