# Supplementary material for: Cosmid based mutagenesis causes genetic instability in Streptomyces coelicolor, as shown by targeting of the lipoprotein signal peptidase gene
Source: Sci Rep. 2016 Jul 12;6:29495. doi: 10.1038/srep29495 (PMC4941574; doi:10.1038/srep29495)
Supplement: Supplementary Information [file srep29495-s1.pdf]

Cosmid based mutagenesis causes genetic instability in *Streptomyces coelicolor*, as shown by targeting of the lipoprotein signal peptidase gene

John T Munnoch[1], David A. Widdick[1,2], Govind Chandra[2], Iain C. Sutcliffe[3], Tracy Palmer[4] and Matthew I Hutchings[1]*

[1]School of Biological Sciences, University of East Anglia, Norwich Research Park, Norwich

[2]Department of Molecular Microbiology, John Innes Centre, Norwich Research Park, Norwich

[3] School of Applied Sciences, Northumbria University, Newcastle

[4] Division of Molecular Microbiology, College of Life Sciences, University of Dundee, Dundee.

[*]Correspondence: M.hutchings@uea.ac.uk

Table S1. Secondary mutations in BJT1004 and its parent wild-type strain relative to the published *Streptomyces coelicolor* genome sequence.

	SNP			GATC[§]		TGAC[§]	
No.	Position[*]	Gene[†]	Change[‡]	M145	BJT1004	M145	BJT1004
1	104233	*sco0124*	---	Y	N	N	N
2	563185	*sco0531*	E128G	Y	N	N	N
3	619172	*sco0577*	---	Y	Y	N	N
4	657081	*sco0617*	E564D	Y	Y	Y	Y
5	1415134	*sco1337*	G15A	Y	Y	Y	N
6	1622503	*sco1516*	Y72N	Y	N	Y	Y
7	1625888	*sco1520*	R13C	Y	Y	Y	Y
8	1634749	*sco1529*	---	Y	Y	Y	Y
9	1642021	-	N/A	N	N	N	Y
10	1644238	*sco1536*	H262Q	Y	N	N	N
11[#]	1644332	*sco1536*	R230P	N	N	Y	Y
12[#]	1644333	*sco1536*	R230G	N	N	Y	Y
13	1649515	-	N/A	N	Y	N	N
14	1740776	-	N/A	N	N	Y	Y
15	2065371	-	N/A	N	N	N	Y
16	2065372	-	N/A	N	N	N	Y
17	2173610	*sco2026*	---	Y	N	N	N
18	2301530	*sco2139*	A249G	N	Y	N	N
19	2976040	*sco2729*	C112G	Y	N	N	N
20	3141224	*sco2886*	W84G	N	Y	N	N
21	3348737	*sco3054*	P36L	Y	Y	N	N
22	3349264	*sco3055*	G101R	Y	Y	Y	N
23	3958102	-	N/A	N	N	Y	N
24	4140160	-	N/A	N	Y	N	N
25	4140161	-	N/A	N	Y	N	N
26	4245354	*sco3860*	V261G	Y	N	N	N
27	4823788	*sco4405*	T219P	Y	N	N	N
28	4863506	-	N/A	Y	N	N	N
29	4863507	-	N/A	Y	N	N	N
30	4868999	-	N/A	Y	N	N	N
31	5016937	*sco4594*	---	N	N	N	Y
32	5018303	*sco4595*	---	N	N	Y	N
33	5044225	*sco4620*	---	N	N	N	Y
34	5095649	*sco4665*	E206A	Y	N	N	N
35	5633841	*sco5182*	R7G	N	N	Y	Y
36	5805609	-	N/A	N	N	N	Y
37	5805610	-	N/A	N	Y	N	Y
38	6367261	-	N/A	N	Y	N	N
39	6890540	*sco6265*	---	N	Y	N	N
40	7044234	*sco6381*	---	Y	Y	Y	Y

41	7118314	*sco6436*	V21L	Y	Y	Y	N
42	7146838	*sco6458*	S224G	Y	Y	N	N
43	7180216	*sco6487*	G263A	Y	Y	Y	Y
44	7213371	*sco6522*	A442G	Y	N	N	N
45	7261050	*sco6560*	L123V	N	N	Y	N
46	7284887	*sco6578*	F83V	Y	Y	N	N
47	7364061	*sco6635*	---	Y	N	Y	N
48	7393003	*sco6657*	R378S	Y	Y	N	N
49	8044092	*sco7236*	---	Y	Y	Y	N
50	8594044	*sco7763*	---	Y	Y	Y	N
51	8612266	-	N/A	Y	N	N	Y

* – SNP base positions within the genome *S. coeliocolor* M145 genome.
† – Annotated *sco* gene number with "-" indicated an intergenic SNP.
‡ – Amino acid change with "---" indicating no change and N/A for intergenic site.
§ – The sequencing round responsible for each result.
# – The combination of SNP 11 and 12 would result in a R230A change.

Table S1. Secondary mutations in BJT1004. Re-sequencing and comparison of the parent strain M145 and the *cis* complemented *lsp* mutant BJT1004 revealed 51 putative single nucleotide polymorphisms (SNPs) between all 4 sequenced samples, of which 13 are unique SNPs detected in at least 1 of the BJT1004 sequences, 4 of these are within coding regions but only 1 SNP was detected in both BJT1004 sequences and is intergenic. One chromosomal rearrangement was detected (see also Figure 2). The final 4 columns indicate the presence of the specific SNP in each sequence with "Y" indicating present and "N" indicating absence. Rows with all samples containing "Y" indicates genomic differences in our lab strain compared to the original genome sequence.

Text S1. Sequence of M145 *sco6811-08* loci.

```
LOCUS       sco6811_08              3658 bp ds-DNA     linear       16-FEB-2016
DEFINITION  .
ACCESSION
VERSION
SOURCE      .
  ORGANISM  .
COMMENT
COMMENT
COMMENT     ApEinfo:methylated:1
FEATURES             Location/Qualifiers
     misc_feature    1..363
                     /label=sco6808
                     /ApEinfo_fwdcolor=#ff0080
                     /ApEinfo_revcolor=green
                     /ApEinfo_graphicformat=arrow_data {{} {0 1 2 0 0 -1} 0}
                     width 5 offset 0
     misc_feature    502..1731
                     /label=sco6809
                     /ApEinfo_fwdcolor=#ffff00
                     /ApEinfo_revcolor=green
                     /ApEinfo_graphicformat=arrow_data {{} {0 1 2 0 0 -1} 0}
                     width 5 offset 0
     misc_feature    1738..2196
                     /label=sco6810
                     /ApEinfo_fwdcolor=#ff8000
                     /ApEinfo_revcolor=green
                     /ApEinfo_graphicformat=arrow_data {{} {0 1 2 0 0 -1} 0}
                     width 5 offset 0
     misc_feature    2264..3658
                     /label=sco6811
                     /ApEinfo_fwdcolor=#00f000
                     /ApEinfo_revcolor=green
                     /ApEinfo_graphicformat=arrow_data {{} {0 1 2 0 0 -1} 0}
                     width 5 offset 0
     misc_feature    395..583
                     /label=scr6809
                     /ApEinfo_fwdcolor=#ff0000
                     /ApEinfo_revcolor=green
                     /ApEinfo_graphicformat=arrow_data {{0 1 2 0 0 -1} {} 0}
                     width 5 offset 0
ORIGIN
        1 tcacagttct ttcgttgcct ggggtgcagg gtccaggatg gagcggatcc ggcgcagtcc
       61 ttcgtggttg acggagaacc aggcccaagt accgcgccgt tcccggtcca ggagccctgc
      121 ttcagtcatg atcttcaagt ggtgactgac ggtggactgg cgcagcccca gactgtcggc
      181 cagatcacag acgcaggcct cgccatcggg ggcctgttcc aggagtcgaa acaattgcag
      241 gcgggtgggg tcggcgatgg atctcagtat cgtggacagc tgttcggctt cggatcgctc
      301 cagaggctga cagttcagcc ctggcccgca cgagccacct tcgaggaacc cgtcatccga
      361 catctgcaga tctatgacag tcacccgtca accatagacg cagggtgatc accttgacac
      421 gatcgaatat ggccggactg cgagttcacc tgatgttggg cagccgctag gacaggcggc
      481 ctccgcgtcg gtccttgttg gctatggctt tctggcagcc ggagtcggtg ccgtgtacca
      541 ggcgatgagc gctgcggcgg ccgaggccaa cgcgagagcg gagaacaggc gggggtagcc
      601 gccgagaggt gcggccaggg cggtgccagc gaacggagcg agtgcggagg cggtcgtcgt
      661 gggggcggcg agaaggccgg agaggcggcc gtagcgatgg gcgccccatc ggtctgtgat
      721 ggcggtggcc tgcagcaggg tcaggttgcc gcggacagta ccggcgatga cggccagcgc
      781 cgcaagcagc ccgtagggtc cgggaatgag cgcgaaggct gcggtggtca cccctcccat
      841 gccgatcagc actgctgcgc gcagagcggg ggagctgcgg cgggctagtg gggcgtagag
      901 ggtgcggccg agggtctggc cggctccgcc cagccccaga gcccaggcgg cttcggcagc
      961 ggtgtagccg cggtcgagta gcagggggac gagtgtgatg acgacggcgt atgtcgcgaa
     1021 cgccgaaaga gtcagagccg ttgccagaag aaggaaggga cggctacgtg tgatgacggt
     1081 cgtagacgcg gtctcacctg ttgtgcttgg gggcggggcg gggggccatg ggccgcgcag
     1141 tgcgtaggcg tggacgggca ccgtcactgt ggccaggacg agagccagca gcaggtaggt
     1201 gtgccgccaa ctgaagtggt tggccaactg ggcggtgagg ggtgcgaaga cggtcgatgc
     1261 caggccgccg gcgagagtga cgatcgtcag cgcacgtacg tggttggggg cccaccagcg
     1321 ggtcaatgcc gcgaaggcgg gctggtagaa ggtggaggcc atggcgaacc ccgccagtag
     1381 ccatccggcg gtgaacacgg tcaggttggg ggcgctggcg atgatgacca agctgactgt
     1441 tccaaccacc gaaccgacgg tcatgactgt gcgagggcct cgggcatcga ggatgcggcc
     1501 gatgcgaatg ccggcaacgg cggacatcag caaggcagcg gagaacgcgg ctgtggtcgc
```

```
     1561 agtggcgctc cagccggtgg ccatggtgat ctgtggattg aggacgggaa aggcgtagta
     1621 gacgatgccc cagctggtga tctgggtcag acacagggcg gggagggccg cccgcggccg
     1681 cgtcggctgg ttgagggcgg ccgcggggga ggccatggag cggtagctca tgccttctca
     1741 gcaacaactg gttgtggtgg cctgcggtcc cggttggacg gcgtcggtgg gtgcgcaaca
     1801 cgtgctgccg ggttgcttgg ctagggtgtc ggcgtccgcc ttcacgacgt acacctccca
     1861 cgcctcctga ccggggccgt gcacccacac cttgtcctgc agggcgtagc agcacgcggt
     1921 gtcgttctct tcgcgagtgc tcagccctgc ctggctcagc cgggcggtgg ctgccgcgac
     1981 gtcctcggtg gatgccacct cgacaccgag gtggtccagg cgggtgtccg tgccctcttc
     2041 tccctcgatc agaacgagct tgagcggcgg ctcggtgacg gcgaagttcg catagccatc
     2101 acggagcttg gcaggctcgg tgtcgaggag tttgctgtag aaggcgatgg ctgactgcaa
     2161 gtcgggaact cgcagggcga gttgtatccg tgacatgggg cattcctcct gccggttcag
     2221 tggtgcaggc tcccgcggcc gcaacggctg cgggtgggga cgatcaggcg ttgcagctgc
     2281 tgccgggtgt cgtggccggt ccgggagaga tggtggacaa ctggaccagc tgcggagcgg
     2341 acggagcgca gcagccttcc ttttcgggct cggcggcgcc gtccgtgtcg aagagtccgg
     2401 agcctccgca cacgccggtt tccggcagga ccagttcgac gcggtcggca gcttcggttt
     2461 cgccggccag ggcggccgcg atcgagcgga cctgctcgta gccggtcatc gccaggaacg
     2521 tgggagctcg tccgtagctc ttcatgccga ccaggtagac accggagtcc gggtgggaca
     2581 gttcccgatg gccgtgcggg tagacggttc cgcacgagtg ctggttgggg tcgatcagcg
     2641 gagcgagctc gaccggtgcc tgcaggcgct cgtccaggcg caggcggacc tcggagagga
     2701 aggacaggtc ggggcggaaa ccagtcagga caatgacctc gtccactgcc tggagccgtc
     2761 gtccgtcctc cgcgacgagg atgagccggt cctcagcacc gcgttctacg gcctcggtgc
     2821 gaaagccggt gaccgcctcg gcgtggccct catcgacggc ggccttggca gccagaccga
     2881 gggcaccgcg tgcgggcagc tggtccgctt caccgccgcc gtaggtggag cccgagaggc
     2941 cgcggcgcag tacccatacc gtctttgtcg ttgcgctgtt ctgcgatcgc gcgagttcgg
     3001 ccagtgaggc gagtgcggtg aatgccgagg cgcccgagcc gatgaccgcg gtgcgcttct
     3061 ccgcgtagcg tgcgcgcacc gccgggtcgg tgaggtcggg gacgcggtag gtgacatggt
     3121 cggaggccat gtgctcgccg agggcgggta gaccgctgct gccggcgggg gagggcaggg
     3181 accaggtgcc ggaggcatcg atcaccgcag aggcggtgat cctttcttcc cggccgtctg
     3241 tgtgcttcac ctggacgacg tacggctgtg cgtcgcggtc ggcgtccacg acgcggtccc
     3301 ggccggcctt cgagacgccg gtgactcggg ctccgtagcg gatctggtcg ccgagggtgt
     3361 cggcaagcgg ctgcaggtag tgcttcaccc agtcgccgcc gctggggtag gtgctgctgt
     3421 cgggcctgct ccacccggtg gggtgagga gcttttcggc ggcggggtcc acgacctcgc
     3481 cccaggtgga gaacaggcgg acgtgtgccc actcgctgac ggcgctgccg gcggtggggc
     3541 ctgcttccag cacgagcggg cgcagacccc gttccagcag atgagctgcg gcagccagcc
     3601 caatgggtcc ggcgccgatg acgactacgg gcggggtgag gacggcgggc gcagtcac
//
```

Text S1. **Sequence of M145 *sco6811-08* loci.** Genbank (.gbk) formatted sequence file containing sequence information of the wildtype *sco6811-08* region [29].

Table S2. List of genes (and their functions) on cosmid St4A10

Sco number	Gene name	Protein function
sco2068		Hypothetical
sco2069		Hypothetical
sco2070		Putative membrane protein
sco2071		Putative antiporter
sco2072		Hypothetical
sco2073		Possible ribosomal large subunit pseudouridine synthase
sco2074	*lsp*	Lipoprotein signal peptidase
sco2075		Putative DNA binding protein
sco2076		Probable isoleucyl-tRNA synthetase
sco2077	*divIVA*	Division site selection protein
sco2078		Putative membrane protein
sco2079		Conserved hypothetical protein
sco2080		Conserved hypothetical protein
sco2081		Conserved hypothetical protein
sco2082	*ftsZ*	Cell division protein, forms septal ring
sco2083	*ftsQ*	Required for efficient sporulation, but not growth and viability
sco2084	*murG*	Generates lipid II
sco2085	*ftsW*	Flippase for lipid II.
sco2086	*murD*	Adds second amino acid to the growing pentapeptide chain on cell wall precursor (in the cytoplasm)
sco2087	*murX* (*mraY*)	Generates lipid I
sco2088	*murF*	Adds D-Ala-D-Ala dipeptide to complete the pentapeptide chain on cell wall precursor (in the cytoplasm)
sco2089	*murE*	Adds third amino acid to the growing pentapeptide chain on cell wall precursor (in the cytoplasm)
sco2090	*ftsI*	PBP3 - transpeptidase involved in cell division, interacts with FtsW
sco2091	*ftsL*	Possible membrane protein
sco2092		Conserved hypothetical protein
sco2093		Conserved hypothetical protein
sco2094		Transcription factor
sco2095		Putative membrane protein
sco2096		Putative membrane protein
sco2097		Putative membrane protein
sco2098		Possible methyltransferase
sco2099		Hypothetical
sco2100		Transcription factor
sco2101		Transposon
sco2102		Putative membrane protein
sco2103	*metF*	5,10-methylenetetrahydrofolate reductase
sco2104		possible thiamin phosphate pyrophosphorylase

Table S2. List of genes (and their functions) on cosmid St4A10.

Text S2. Sequence of BJT1004 *sco6811-08* loci.

```
LOCUS       Text_S4_BJT1004_        6028 bp ds-DNA     linear       16-FEB-2016
DEFINITION  .
ACCESSION   
VERSION     
SOURCE      .
  ORGANISM  .
COMMENT     
COMMENT     ApEinfo:methylated:1
FEATURES             Location/Qualifiers
     misc_feature    1..363
                     /label=sco6808
                     /ApEinfo_fwdcolor=#ff0080
                     /ApEinfo_revcolor=green
                     /ApEinfo_graphicformat=arrow_data {{0 1 2 0 0 -1} {} 0}
                     width 5 offset 0
     misc_feature    2872..4101
                     /label=sco6809
                     /ApEinfo_fwdcolor=#ffff00
                     /ApEinfo_revcolor=green
                     /ApEinfo_graphicformat=arrow_data {{0 1 2 0 0 -1} {} 0}
                     width 5 offset 0
     misc_feature    4108..4566
                     /label=sco6810
                     /ApEinfo_fwdcolor=#ff8000
                     /ApEinfo_revcolor=green
                     /ApEinfo_graphicformat=arrow_data {{0 1 2 0 0 -1} {} 0}
                     width 5 offset 0
     misc_feature    4634..6028
                     /label=sco6811
                     /ApEinfo_fwdcolor=#00f000
                     /ApEinfo_revcolor=green
                     /ApEinfo_graphicformat=arrow_data {{0 1 2 0 0 -1} {} 0}
                     width 5 offset 0
     misc_feature    501..1964
                     /label=SCO6393 (IS21)
                     /ApEinfo_fwdcolor=cyan
                     /ApEinfo_revcolor=green
                     /ApEinfo_graphicformat=arrow_data {{0 1 2 0 0 -1} {} 0}
                     width 5 offset 0
     misc_feature    1961..2731
                     /label=SCO6394 (IS21)
                     /ApEinfo_fwdcolor=#00ff00
                     /ApEinfo_revcolor=green
                     /ApEinfo_graphicformat=arrow_data {{0 1 2 0 0 -1} {} 0}
                     width 5 offset 0
     misc_feature    join(2787..2871,395..416)
                     /label=scr6809
                     /ApEinfo_fwdcolor=#ff0000
                     /ApEinfo_revcolor=green
                     /ApEinfo_graphicformat=arrow_data {{0 1 2 0 0 -1} {} 0}
                     width 5 offset 0
     misc_feature    188..207
                     /label=JTM07
                     /ApEinfo_fwdcolor=cyan
                     /ApEinfo_revcolor=green
                     /ApEinfo_graphicformat=arrow_data {{0 1 2 0 0 -1} {} 0}
                     width 5 offset 0
     misc_feature    3053..3071
                     /label=JTM08
                     /ApEinfo_fwdcolor=cyan
                     /ApEinfo_revcolor=green
                     /ApEinfo_graphicformat=arrow_data {{0 1 2 0 0 -1} {} 0}
                     width 5 offset 0
ORIGIN
        1 tcacagttct ttcgttgcct ggggtgcagg gtccaggatg gagcggatcc ggcgcagtcc
       61 ttcgtggttg acggagaacc aggcccaagt accgcgccgt tcccggtcca ggagccctgc
      121 ttcagtcatg atcttcaagt ggtgactgac ggtggactgg cgcagcccca gactgtcggc
```

181 cagatcacag acgcaggcct cgccatcggg ggcctgttcc aggagtcgaa acaattgcag
241 gcgggtgggg tcggcgatgg atctcagtat cgtggacagc tgttcggctt cggatcgctc
301 cagaggctga cagttcagcc ctggcccgca cgagccacct tcgaggaacc cgtcatccga
361 catctgcaga tctatgacag tcacccgtca accatagacg cagggtgatc accttgACAC
421 Gtgtcggcgt acaagcgaac gtgcaccacc tcgtacggat gaaagtgcac cattcctgat
481 gcggtgacgg ggtgtcggcc gtgagtctgc tgctgtcggt tcggggcggc agggaggcac
541 tgacggtggt cttggatccg catcgctggc tggagttgag gcgatttcgt ccgctgtacg
601 agtcaggtgc gatgagcctg cgggagatcg cgaaggagac cgggctgaac cgccggacgg
661 tcagcaagta cctcaaggac ccggcctcgc tcgcgccgcc gaagagagaa gtcgcagatc
721 agcggcctcg acgggtggtg gacgaggtgg cgccgctgat cgacgcaatg ctcagatccg
781 agatcctgct caagggaagg gtgatccacg agcgcctggt ccaggagtac ggcgtcgcga
841 tcaactatca acgggtgaag ctatatctgc aagaagcccg gccccggatc gcggaggaac
901 tgggtatcag cccgggcgag ttggcgggtc tgcaccggcg gttcgaggtc gtcccgggtg
961 ctcaggctca agtcgactgg ggggacgagg gcaagatcct cgcccatgtc ggcatcccga
1021 aggtctactc cttccacatg acgctgtcgt actcgcgcga cccgttctgc tgcttcacca
1081 ccagccagga cttggcaacg ttcttcgact gccaccgtaa ggcgttcgcg cacttcggcg
1141 gggtgccgat gagcgttgtc tacgaccgca cgaagacggt cgtgcgccgg cacgtcgccc
1201 ccggggaggc ggttccgctg catccggagg cggtcgcctt cgccgggcac tacgacttcg
1261 acatcgacgt gctggccgcc taccgcccgc agggcaaggg ccgggtcgag cggcaggtcg
1321 gcatcgtccg cgaccacgtc ctggccggcc gggccttctc ctcggtcgaa gagatgaacg
1381 ccgccttcgc ggcctgggtg ccgttccggc gggcgaaggt ccacggcacc cacggtgaag
1441 tcatcgggca ccgggccgtg cgcgatcaca tggccctccg cccactgcca cggacccccct
1501 atgtggtcgc ccagcggcat ctgcggcacg tcggcaagga ctgcctggtc gcctttgacg
1561 ccaacctcta ctcggtgccc gcccgcaaga tccgcccccg ccagctggtc gagatccggg
1621 ccacgaagtc gcaggtcagc ctgcactcca ccgtccctga cccaagtggc cggaccttgt
1681 tggccgtcca tcctcgggcc gttggccgag gtgcacgcat cgtggatgag acgcactggg
1741 acggcctgcc caccggcgct ggccggcgcg tcaccaccgg cgacgctctg ccctcgcccc
1801 gccgcggcca gccggccggg ccggagaccg gaccgcttca ggctctgctg aaccgagccg
1861 ccgccgcgaa cgtcgaggtc ggccgccgac cgctgtcggt ctatgacgag ctgaccggca
1921 cccgtccctt caccgcaccc gccccgacca aggaagcccg ttgagcgagc tgaccagcaa
1981 ccgcatccgc accaccgccg ccaagctcgg cctgccgcac ctggccgagg ccctcaacca
2041 atacgtccag cgggcggacg aggccaagat gggctacctc gacttcctcg acttggtgct
2101 ggctgaggaa ctcgccgtcc gtgacgaccg gcgcttccgc aacggcctgc ggctgtcgaa
2161 gctgccgcac cacaagacac tggaggacta cgacttctcc ttccagcccg acctcgatcc
2221 gcgcaaggtc aaggacctgg ccaccctctc cttcatcgag gacaaggcca atgtcgctct
2281 gctcgggccg cccggggtcg gcaagacgca catcgccgtc gccctcgcgg tcgcggcctg
2341 ccgggccggg tactcgatct acttcaccag cctcgacgac atggtccgcc acctcaaagc
2401 cgccgaggac cagggccggc tgatcagcaa gctcaccagc taccttcgcc ccgccgttct
2461 cgtcgtcgac gaggtgggct accaaccgct tgagcgggcc gaggcgaacc tggtctttca
2521 ggtcatctcc aagcgttacg agaagggctc catcatcctg acctcgaaca agaccttcgg
2581 tgagtggggc caggtctttg gcgacgaggt cctggccacc gcgatcctcg accgcctcct
2641 gcaccactgc gaagtcgtct cgatcaacgg caacagttac cggctcaaga accgcctcca
2701 ggccatcgaa cgagacaccg acgtggcctg agcagtggtg cacgtaactt cgtactcggt
2761 ggtgcactca gacgagtacc tggacaacac gatcgaatat ggccggactg cgagttcacc
2821 tgatgttggg cagccgctag gacaggcggc ctccgcgtcg gtccttgttg gctatggctt
2881 tctggcagcc ggagtcggtg ccgtgtacca ggcgatgagc gctgcggcgg ccgaggccaa
2941 cgcgagagcg gagaacaggc gggggtagcc gccgagaggt gcggccaggg cggtgccagc
3001 gaacggagcg agtgcggagg cggtcgtcgt gggggcggcg agaaggccgg agaggcggcc
3061 gtagcgatgg gcgcccccatc ggtctgtgat ggcggtggcc tgcagcaggg tcaggttgcc
3121 gcggacagta ccggcgatga cggccagcgc cgcaagcagc ccgtagggtc cgggaatgag
3181 cgcgaaggct gcggtggtca cccctcccat gccgatcagc actgctgcgc gcagagcggg
3241 ggagctgcgg cgggctagtg gggcgtagag ggtgcggccg agggtctggc cggctccgcc
3301 cagccccaga gcccaggcgg cttcggcagc ggtgtagccg cggtcgagta gcagggggac
3361 gagtgtgatg acgacggcgt atgtcgcgaa cgccgaaaga gtcagagccg ttgccagaag
3421 aaggaaggga cggctacgtg tgatgacggt cgtagacgcg gtctcacctg ttgtgcttgg
3481 gggcggggcg gggggccatg ggccgcgcag tgcgtaggcg tggacgggca ccgtcactgt
3541 ggccaggacg agagccagca gcaggtaggt gtgccgccaa ctgaagtggt tggccaactg
3601 ggcggtgagg ggtgcgaaga cggtcgatgc caggccgccg gcgagagtga cgatcgtcag
3661 cgcacgtacg tggttggggg cccaccagcg ggtcaatgcc gcgaaggcgg gctggtagaa
3721 ggtggaggcc atggcgaacc ccgccagtag ccatccggcg gtgaacacgg tcaggttggg
3781 ggcgctggcg atgatgacca agctgactgt tccaaccacc gaaccgacgg tcatgactgt
3841 gcgagggcct cgggcatcga ggatgcggcc gatgcgaatg ccggcaacgg cggacatcag
3901 caaggcagcg gagaacgcgg ctgtggtcgc agtggcgctc cagccggtgg ccatggtgat
3961 ctgtggattg aggacgggaa aggcgtagta gacgatgccc cagctggtga tctgggtcag
4021 acacagggcg gggagggccg cccgcggccg cgtcggctgg ttgagggcgg ccgcgggggga
4081 ggccatggag cggtagctca tgccttctca gcaacaactg gttgtggtgg cctgcggtcc
4141 cggttggacg gcgtcggtgg gtgcgcaaca cgtgctgccg ggttgcttgg ctagggtgtc
4201 ggcgtccgcc ttcacgacgt acacctccca cgcctcctga ccggggccgt gcacccacac

```
     4261 cttgtcctgc agggcgtagc agcacgcggt gtcgttctct tcgcgagtgc tcagccctgc
     4321 ctggctcagc cgggcggtgg ctgccgcgac gtcctcggtg gatgccacct cgacaccgag
     4381 gtggtccagg cgggtgtccg tgccctcttc tccctcgatc agaacgagct tgagcggcgg
     4441 ctcggtgacg gcgaagttcg catagccatc acggagcttg gcaggctcgg tgtcgaggag
     4501 tttgctgtag aaggcgatgg ctgactgcaa gtcgggaact cgcagggcga gttgtatccg
     4561 tgacatgggg cattcctcct gccggttcag tggtgcaggc tcccgcggcc gcaacggctg
     4621 cgggtgggga cgatcaggcg ttgcagctgc tgccgggtgt cgtggccggt ccgggagaga
     4681 tggtggacaa ctggaccagc tgcggagcgg acggagcgca gcagccttcc ttttcgggct
     4741 cggcggcgcc gtccgtgtcg aagagtccgg agcctccgca cacgccggtt tccggcagga
     4801 ccagttcgac gcggtcggca gcttcggttt cgccggccag ggcggccgcg atcgagcgga
     4861 cctgctcgta gccggtcatc gccaggaacg tgggagctcg tccgtagctc ttcatgccga
     4921 ccaggtagac accggagtcc gggtgggaca gttcccgatg gccgtgcggg tagacggttc
     4981 cgcacgagtg ctggttgggg tcgatcagcg gagcgagctc gaccggtgcc tgcaggcgct
     5041 cgtccaggcg caggcggacc tcggagagga aggacaggtc ggggcggaaa ccagtcagga
     5101 caatgacctc gtccactgcc tggagccgtc gtccgtcctc cgcgacgagg atgagccggt
     5161 cctcagcacc gcgttctacg gcctcggtgc gaaagccggt gaccgcctcg gcgtggccct
     5221 catcgacggc ggccttggca gccagaccga gggcaccgcg tgcgggcagc tggtccgctt
     5281 caccgccgcc gtaggtggag cccgagaggc cgcggcgcag tacccatacc gtctttgtcg
     5341 ttgcgctgtt ctgcgatcgc gcgagttcgg ccagtgaggc gagtgcggtg aatgccgagg
     5401 cgcccgagcc gatgaccgcg gtgcgcttct ccgcgtagcg tgcgcgcacc gccgggtcgg
     5461 tgaggtcggg gacgcggtag gtgacatggt cggaggccat gtgctcgccg agggcgggta
     5521 gaccgctgct gccggcgggg gagggcaggg accaggtgcc ggaggcatcg atcaccgcag
     5581 aggcggtgat cctttcttcc cggccgtctg tgtgcttcac ctggacgacg tacggctgtg
     5641 cgtcgcggtc ggcgtccacg acgcggtccc ggccggcctt cgagacgccg gtgactcggg
     5701 ctccgtagcg gatctggtcg ccgagggtgt cggcaagcgg ctgcaggtag tgcttcaccc
     5761 agtcgccgcc gctggggtag gtgctgctgt cgggcctgct ccacccggtg gggtgagga
     5821 gcttttcggc ggcggggtcc acgacctcgc cccaggtgga gaacaggcgg acgtgtgccc
     5881 actcgctgac ggcgctgccg gcggtggggc ctgcttccag cacgagcggg cgcagacccc
     5941 gttccagcag atgagctgcg gcagccagcc caatgggtcc ggcgccgatg acgactacgg
     6001 gcggggtgag gacggcgggc gcagtcac
//
```

Text S2. Sequence of BJT1004 *sco6811-08* loci. Genbank (.gbk) formatted sequence file containing sequence information of the BJT1004 *sco6811-08* loci following sequencing with primers JM0093 and JM0094. WT sequence information was updated using the resulting sequences produced with the noted primers.

Text S3. Sequence of pJM017 insert (pMC500 derivative with *scr6809*), attempt at overexpression.

```
LOCUS       pMC500_modified_         636 bp ds-DNA     linear       16-FEB-2016
DEFINITION  .
ACCESSION
VERSION
SOURCE      .
  ORGANISM  .
COMMENT
COMMENT     ApEinfo:methylated:1
FEATURES             Location/Qualifiers
     misc_feature    631..636
                     /label=hindIII
                     /ApEinfo_fwdcolor=cyan
                     /ApEinfo_revcolor=green
                     /ApEinfo_graphicformat=arrow_data {{0 1 2 0 0 -1} {} 0}
                     width 5 offset 0
     misc_feature    625..630
                     /label=BglII
                     /ApEinfo_fwdcolor=#ffff00
                     /ApEinfo_revcolor=green
                     /ApEinfo_graphicformat=arrow_data {{0 1 2 0 0 -1} {} 0}
                     width 5 offset 0
     misc_feature    582..624
                     /label=tmmr(rev) (terminator)
                     /ApEinfo_fwdcolor=#c0c0c0
                     /ApEinfo_revcolor=green
                     /ApEinfo_graphicformat=arrow_data {{0 1 2 0 0 -1} {} 0}
                     width 5 offset 0
     misc_feature    576..581
                     /label=BamHI
                     /ApEinfo_fwdcolor=cyan
                     /ApEinfo_revcolor=green
                     /ApEinfo_graphicformat=arrow_data {{0 1 2 0 0 -1} {} 0}
                     width 5 offset 0
     misc_feature    387..575
                     /label=scr6809
                     /ApEinfo_fwdcolor=#ffff00
                     /ApEinfo_revcolor=green
                     /ApEinfo_graphicformat=arrow_data {{0 1 2 0 0 -1} {} 0}
                     width 5 offset 0
     misc_feature    381..386
                     /label=BamHI(1)
                     /ApEinfo_label=BamHI
                     /ApEinfo_fwdcolor=cyan
                     /ApEinfo_revcolor=green
                     /ApEinfo_graphicformat=arrow_data {{0 1 2 0 0 -1} {} 0}
                     width 5 offset 0
     misc_feature    375..380
                     /label=EcoRV
                     /ApEinfo_fwdcolor=#ff80ff
                     /ApEinfo_revcolor=green
                     /ApEinfo_graphicformat=arrow_data {{0 1 2 0 0 -1} {} 0}
                     width 5 offset 0
     misc_feature    369..374
                     /label=EcoRI
                     /ApEinfo_fwdcolor=#ff8040
                     /ApEinfo_revcolor=green
                     /ApEinfo_graphicformat=arrow_data {{0 1 2 0 0 -1} {} 0}
                     width 5 offset 0
     misc_feature    100..368
                     /label=ermEp*
                     /ApEinfo_fwdcolor=#0000ff
                     /ApEinfo_revcolor=green
                     /ApEinfo_graphicformat=arrow_data {{0 1 2 0 0 -1} {} 0}
                     width 5 offset 0
     misc_feature    94..99
                     /label=XbaI
                     /ApEinfo_fwdcolor=cyan
                     /ApEinfo_revcolor=green
                     /ApEinfo_graphicformat=arrow_data {{0 1 2 0 0 -1} {} 0}
                     width 5 offset 0
     misc_feature    19..93
                     /label=to (terminator)
                     /ApEinfo_fwdcolor=#008000
                     /ApEinfo_revcolor=green
```

```
                     /ApEinfo_graphicformat=arrow_data {{0 1 2 0 0 -1} {} 0}
                     width 5 offset 0
     misc_feature    13..18
                     /label=KpnI
                     /ApEinfo_fwdcolor=#80ff00
                     /ApEinfo_revcolor=green
                     /ApEinfo_graphicformat=arrow_data {{0 1 2 0 0 -1} {} 0}
                     width 5 offset 0
     misc_feature    7..12
                     /label=bglII
                     /ApEinfo_fwdcolor=#ffff00
                     /ApEinfo_revcolor=green
                     /ApEinfo_graphicformat=arrow_data {{0 1 2 0 0 -1} {} 0}
                     width 5 offset 0
     misc_feature    1..6
                     /label=NsiI
                     /ApEinfo_fwdcolor=cyan
                     /ApEinfo_revcolor=green
                     /ApEinfo_graphicformat=arrow_data {{0 1 2 0 0 -1} {} 0}
                     width 5 offset 0
ORIGIN
        1 ATGCATAGAT CTGGTACCTC CAGTAATGAC CTCAGAACTC CATCTGGATT TGTTCAGAAC
       61 GCTCGGTTGC CGCCGGGCGT TTTTTATTGG TGATCTAGAA GCCCGACCCG AGCACGCGCC
      121 GGCACGCCTG GTCGATGTCG GACCGGAGTT CGAGGTACGC GGCTTGCAGG TCCAGGAAGG
      181 GGACGTCCAT GCGAGTGTCC GTTCGAGTGG CGGCTTGCGC CCGATGCTAG TCGCGGTTGA
      241 TCGGCGATCG CAGGTGCACG CGGTCGATCT TGACGGCTGG CGAGAGGTGC GGGGAGGATC
      301 TGACCGACGC GGTCCACACG TGGCACCGCG ATGCTGTTGT GGGCACAATC GTGCCGGTTG
      361 GTAGGATCGA ATTCGATATC GGATCCCTCC GCTCTCGCGT TGGCCTCGGC CGCCGCAGCG
      421 CTCATCGCCT GGTACACGGC ACCGACTCCG GCTGCCAGAA AGCCATAGCC AACAAGGACC
      481 GACGCGGAGG CCGCCTGTCC TAGCGGCTGC CCAACATCAG GTGAACTCGC AGTCCGGCCA
      541 TATTCGATCG TGTCAAGGTG ATCACCCTGC GTCTAGGATC CAGAAGCAGG GGCCCCGACC
      601 GTGTCGGGGC CCCTGCACGG GTTGAGATCT AAGCTT
//
```

Text S3. Sequence of pJM017 insert (pMC500 derivative with *scr6809*). This insert sequence was synthesized by Genscript and cloned into pMS82.

Text S4. Sequence results of JTM018.01, combined sequences from JM0150 and JM0151. PCR product size was ~1.4kb with the sequence results covering 1362 bp overlapping in the centre.

```
LOCUS       New_DNA                 1362 bp ds-DNA     linear       16-FEB-2016
DEFINITION  .
ACCESSION
VERSION
SOURCE      .
  ORGANISM  .
COMMENT
COMMENT     ApEinfo:methylated:1
FEATURES             Location/Qualifiers
     misc_feature    473..1362
                     /label=lspTR sequence
                     /ApEinfo_fwdcolor=cyan
                     /ApEinfo_revcolor=green
                     /ApEinfo_graphicformat=arrow_data {{0 1 2 0 0 -1} {} 0}
                     width 5 offset 0
     misc_feature    1..960
                     /label=LspTF sequence
                     /ApEinfo_fwdcolor=#00ff00
                     /ApEinfo_revcolor=green
                     /ApEinfo_graphicformat=arrow_data {{0 1 2 0 0 -1} {} 0}
                     width 5 offset 0
ORIGIN
        1 tgtaggctgg agctgcttcg aagttcctat actttctaga gaataggaac ttcggaatag
       61 gaacttatga gctcagccaa tcgactggcg agcggcatcg cattcttcgc atcccgcctc
      121 tggcggatgc aggaagatca acggatctcg gcccagttga cccagggctg tcgccacaat
      181 gtcgcgggag cggatcaacc gagcaaaggc atgaccgact ggaccttcct tctgaaggct
      241 cttctccttg agccacctgt ccgccaaggc aaagcgctca cagcagtggt cattctcgag
      301 ataatcgacg cgtaccaact tgccatcctg aagaatggtg cagtgtctcg gcaccccata
      361 gggaaccttt gccatcaact cggcaagatg cagcgtcgtg ttggcatcgt gtcccacgcc
      421 gaggagaagt acctgcccat cgagttcatg gacacgggcg accgggcttg caggcgagtg
      481 aggtggcagg ggcaatggat cagagatgat ctgctctgcc tgtggccccg ctgccgcaaa
      541 ggcaaatgga tgggcgctgc gctttacatt tggcaggcgc cagaatgtgt cagagacaac
      601 tccaaggtcc ggtgtaacgg gcgacgtggc aggatcgaac ggctcgtcgt ccagacctga
      661 ccacgagggc atgacgagcg tccctcccgg acccagcgca gcacgcaggg cctcgatcag
      721 tccaagtggc ccatcttcga ggggccggac gctacggaag gagctgtgga ccagcagcac
      781 accgccgggg gtaaccccaa ggttgagaag ctgaccgatg agctcggctt ttcgccattc
      841 gtattgcacg acattgcact ccaccgctga tgacatcagt cgatcatagc acgatcaacg
      901 gcactgttgc aaatagtcgg tggtgataaa cttatcatcc cctttgctg atggagctgc
      961 acatgaaccc attcaaaggc cggcattttc agcgtgacat cattctgtgg gccgtacgct
     1021 ggtactgcaa atacggcatc agttaccgtg agctgcattt tccgctgcat aaccctgctt
     1081 cggggtcatt atagcgattt tttcggtata tccatccttt ttcgcacgat atacaggatt
     1141 ttgccaaagg gttcgtgtag actttccttg gtgtatccaa cggcgtcagc cgggcaggat
     1201 aggtgaagta ggcccacccg cgagcgggtg ttccttcttc actgtccctt attcgcacct
     1261 ggcggtgctc aacgggaatc ctgctctgcg aggctggcgg gaacttcgaa gttcctatac
     1321 tttctagaga ataggaactt cgaactgcag gtcgacggat cc
//
```

Text S4. Sequence results of JTM018.01 (*lsp* mutant produced with the *lsp* suicide vector pJM016), with combined sequences from JM0150 and JM0151. PCR product size was ~1.4kb was produced using the noted primers and sequenced in both directions, results covering 1362 bp of this product with the original sequence files overlapping in the centre.

Text S5. An example of the expected results following insertion of pJM016 into the M145 chromosome.

```
LOCUS       pLAS_insertion_i        5497 bp ds-DNA     linear       16-FEB-2016
DEFINITION  .
ACCESSION
VERSION
SOURCE      .
  ORGANISM  .
COMMENT
COMMENT
COMMENT     ApEinfo:methylated:1
FEATURES             Location/Qualifiers
     misc_feature    5478..5497
                     /label=JM0151 (Sco Lsp Test Rev)
                     /ApEinfo_fwdcolor=cyan
                     /ApEinfo_revcolor=green
                     /ApEinfo_graphicformat=arrow_data {{0 1 2 0 0 -1} {} 0}
                     width 5 offset 0
     misc_feature    1..20
                     /label=JM0150 (Sco Lsp Test For)
                     /ApEinfo_fwdcolor=cyan
                     /ApEinfo_revcolor=green
                     /ApEinfo_graphicformat=arrow_data {{0 1 2 0 0 -1} {} 0}
                     width 5 offset 0
     misc_feature    join(4997..5461,37..186)
                     /label=lsp
                     /ApEinfo_fwdcolor=#00ff00
                     /ApEinfo_revcolor=green
                     /ApEinfo_graphicformat=arrow_data {{0 1 2 0 0 -1} {} 0}
                     width 5 offset 0
     misc_feature    187..4996
                     /label=pJM016
                     /ApEinfo_fwdcolor=cyan
                     /ApEinfo_revcolor=green
                     /ApEinfo_graphicformat=arrow_data {{0 1 2 0 0 -1} {} 0}
                     width 5 offset 0
ORIGIN
        1 TCGTGCTCAG TCAAGGACCT AGGCTGAGGG ACTCACGTGG CAGAGGCGGA GCGCATCATC
       61 GGTACTCCGG ACATCCCGGA CGCGGCGGGG GAGGGGCAGG AGCGGCCCGA CGCCGACCCG
      121 GAGCGGGAGC AGCAGGAGCA GGAGCAGGCT CCTGAGCGCA CGCGGGGCAA GCGGCGGGTC
      181 GCCGTGCTGT TCGCGGTCGC CCTGTTCGCG TACCTGCTCG ACCTGGGCAG CAAGATGCTG
      241 GTGGTCGCCA AGCTGGAGCA CCACGAGCCG ATCGAGATCA TCGGCGACTG GCTGCGGTTC
      301 GCCGCGATCC GCAACGCGGG CGCGGCCTTC GGCTTCGGCG AGGCGTTCAC GATCATCTTC
      361 ACGGTGATCG CCGCTGCCGT GATCGTCGTG ATCGCCCGCC TCGCGCGCAA GCTGCACAGC
      421 CTGCCCTGGG CGATCGCGCT CGGCCTGCTG CTCGGCGGTG CCCTCGGCAA CCTCACCGAC
      481 CGGATCTTCC GTGCTCCCGG GGTCTTCGAG GGCGCGGTCG TGGACTTCAT CGCCCCCAAG
      541 CACTTCGCCG TCTTCAACCT CGCCGACTCG GCGATCGTGT GCGGCGGCAT CATCACTAGT
      601 GAATTCGCGG CCGCCTGCAG GTCGACCATA TGGGAGAGCT CCCAACGCGT TGGATGCATA
      661 GCTTGAGTAT TCTATAGTGT CACCTAAATA GCTTGGCGTA ATCATGGTCA TAGCTGTTTC
      721 CTGTGTGAAA TTGTTATCCG CTCACAATTC CACACAACAT ACGAGCCGGA AGCATAAAGT
      781 GTAAAGCCTG GGGTGCCTAA TGAGTGAGCT AACTCACATT AATTGCGTTG CGCTCACTGC
      841 CCGCTTTCCA GTCGGGAAAC CTGTCGTGCC AGCTGCATTA ATGAATCGGC CAACGCGCGG
      901 GGAGAGGCGG TTTGCGTATT GGGCGCTCTT CCGCTTCCTC GCTCACTGAC TCGCTGCGCT
      961 CGGTCGTTCG GCTGCGGCGA GCGGTATCAG CTCACTCAAA GGCGGTAATA CGGTTATCCA
     1021 CAGAATCAGG GGATAACGCA GGAAAGAACA TGTGAGCAAA AGGCCAGCAA AAGGCCAGGA
     1081 ACCGTAAAAA GGCCGCGTTG CTGGCGTTTT TCCATAGGCT CCGCCCCCCT GACGAGCATC
     1141 ACAAAAATCG ACGCTCAAGT CAGAGGTGGC GAAACCCGAC AGGACTATAA AGATACCAGG
     1201 CGTTTCCCCC TGGAAGCTCC CTCGTGCGCT CTCCTGTTCC GACCCTGCCG CTTACCGGAT
     1261 ACCTGTCCGC CTTTCTCCCT TCGGGAAGCG TGGCGCTTTC TCATAGCTCA CGCTGTAGGT
     1321 ATCTCAGTTC GGTGTAGGTC GTTCGCTCCA AGCTGGGCTG TGTGCACGAA CCCCCCGTTC
     1381 AGCCCGACCG CTGCGCCTTA TCCGGTAACT ATCGTCTTGA GTCCAACCCG GTAAGACACG
     1441 ACTTATCGCC ACTGGCAGCA GCCACTGGTA ACAGGATTAG CAGAGCGAGG TATGTAGGCG
     1501 GTGCTACAGA GTTCTTGAAG TGGTGGCCTA ACTACGGCTA CACTAGAAGA ACAGTATTTG
     1561 GTATCTGCGC TCTGCTGAAG CCAGTTACCT TCGGAAAAAG AGTTGGTAGC TCTTGATCCG
     1621 GCAAACAAAC CACCGCTGGT AGCGGTGGTT TTTTTGTTTG CAAGCAGCAG ATTACGCGCA
     1681 GAAAAAAAGG ATCTCAAGAA GATCCTTTGA TCTTTTCTAC GGGGTCTGAC GCTCAGTGGA
     1741 ACGAAAACTC ACGTTAAGGG ATTTTGGTCA TGAGATTATC AAAAAGGATC TTCACCTAGA
     1801 TCCTTTTAAA TTAAAAATGA AGTTTTAAAT CAATCTAAAG TATATATGAG TAAACTTGGT
     1861 CTGACAGTTA CCAATGCTTA ATCAGTGAGG CACCTATCTC AGCGATCTGT CTATTTCGTT
```

```
1921 CATCCATAGT TGCCTGACTC CCCGTCGTGT AGATAACTAC GATACGGGAG GGCTTACCAT
1981 CTGGCCCCAG TGCTGCAATG ATACCGCGAG ACCCACGCTC ACCGGCTCCA GATTTATCAG
2041 CAATAAACCA GCCAGCCGGA AGGGCCGAGC GCAGAAGTGG TCCTGCAACT TTATCCGCCT
2101 CCATCCAGTC TATTAATTGT TGCCGGGAAG CTAGAGTAAG TAGTTCGCCA GTTAATAGTT
2161 TGCGCAACGT TGTTGCCATT GCTACAGGCA TCGTGGTGTC ACGCTCGTCG TTTGGTATGG
2221 CTTCATTCAG CTCCGGTTCC CAACGATCAA GGCGAGTTAC ATGATCCCCC ATGTTGTGCA
2281 AAAAAGCGGT TAGCTCCTTC GGTCCTCCGA TCGTTGTCAG AAGTAAGTTG GCCGCAGTGT
2341 TATCACTCAT GGTTATGGCA GCACTGCATA ATTCTCTTAC TGTCATGCCA TCCGTAAGAT
2401 GCTTTTCTGT GACTGGTGAG TACTCAACCA AGTCATTCTG AGAATAGTGT ATGCGGCGAC
2461 CGAGTTGCTC TTGCCCGGCG TCAATACGGG ATAATACCGC GCCACATAGC AGAACTTTAA
2521 AAGTGCTCAT CATTGGAAAA CGTTCTTCGG GGCGAAAACT CTCAAGGATC TTACCGCTGT
2581 TGAGATCCAG TTCGATGTAA CCCACTCGTG CACCCAACTG ATCTTCAGCA TCTTTTACTT
2641 TCACCAGCGT TTCTGGGTGA GCAAAAACAG GAAGGCAAAA TGCCGCAAAA AAGGGAATAA
2701 GGGCGACACG GAAATGTTGA ATACTCATAC TCTTCCTTTT TCAATATTAT TGAAGCATTT
2761 ATCAGGGTTA TTGTCTCATG AGCGGATACA TATTTGAATG TATTTAGAAA AATAAACAAA
2821 TAGGGGTTCC GCGCACATTT CCCCGAAAAG TGCCACCTGA TGCGGTGTGA AATACCGCAC
2881 AGATGCGTAA GGAGAAAATA CCGCATCAGG AAATTGTAAG CGTTAATATT TTGTTAAAAT
2941 TCGCGTTAAA TTTTTGTTAA ATCAGCTCAT TTTTTAACCA ATAGGCCGAA ATCGGCAAAA
3001 TCCCTTATAA ATCAAAAGAA TAGACCGAGA TAGGGTTGAG TGTTGTTCCA GTTTGGAACA
3061 AGAGTCCACT ATTAAAGAAC GTGGACTCCA ACGTCAAAGG GCGAAAAACC GTCTATCAGG
3121 GCGATGGCCC ACTACGTGAA CCATCACCCT AATCAAGTTT TTTGGGGTCG AGGTGCCGTA
3181 AAGCACTAAA TCGGAACCCT AAAGGGAGCC CCCGATTTAG AGCTTGACGG GGAAAGCCGG
3241 CGAACGTGGC GAGAAAGGAA GGGAAGAAAG CGAAAGGAGC GGGCGCTAGG GCGCTGGCAA
3301 GTGTAGCGGT CACGCTGCGC GTAACCACCA CACCCGCCGC GCTTAATGCG CCGCTACAGG
3361 GCGCGTCCAT TCGCCATTCA GGCTGCGCAA CTGTTGGGAA GGGCGATCGG TGCGGGCCTC
3421 TTCGCTATTA CGCCAGCTGG CGAAAGGGGG ATGTGCTGCA AGGCGATTAA GTTGGGTAAC
3481 GCCAGGGTTT TCCCAGTCAC GACGTTGTAA AACGACGGCC AGTGAATTGT AATACGACTC
3541 ACTATAGGGC GAATTGGGCC CGACGTCGCA TGCTCCCGGC CGCCATGGCG GCCGCGGGAA
3601 TTCGATggat cccccgggct gcaggaattc gatgtgtagg ctggagctgc ttcgaagttc
3661 ctatactttc tagagaatag gaacttcgga ataggaactt atgagctcag ccaatcgact
3721 ggcgagcggc atcgcattct tcgcatcccg cctctggcgg atgcaggaag atcaacggat
3781 ctcggcccag ttgacccagg gctgtcgcca caatgtcgcg ggagcggatc aaccgagcaa
3841 aggcatgacc gactggacct tccttctgaa ggctcttctc cttgagccac ctgtccgcca
3901 aggcaaagcg ctcacagcag tggtcattct cgagataatc gacgcgtacc aacttgccat
3961 cctgaagaat ggtgcagtgt ctcggcaccc catagggaac ctttgccatc aactcggcaa
4021 gatgcagcgt cgtgttggca tcgtgtccca cgccgaggag aagtacctgc ccatcgagtt
4081 catggacacg ggcgaccggg cttgcaggcg agtgaggtgg caggggcaat ggatcagaga
4141 tgatctgctc tgcctgtggc cccgctgccg caaaggcaaa tggatgggcg ctgcgcttta
4201 catttggcag gcgccagaat gtgtcagaga caactccaag gtccggtgta acgggcgacg
4261 tggcaggatc gaacggctcg tcgtccagac ctgaccacga gggcatgacg agcgtccctc
4321 ccggacccag cgcagcacgc agggcctcga tcagtccaag tggcccatct tcgaggggcc
4381 ggacgctacg gaaggagctg tggaccagca gcacaccgcc gggggtaacc ccaaggttga
4441 gaagctgacc gatgagctcg gcttttcgcc attcgtattg cacgacattg cactccaccg
4501 ctgatgacat cagtcgatca tagcacgatc aacggcactg ttgcaaatag tcggtggtga
4561 taaacttatc atcccctttt gctgatggag ctgcacatga acccattcaa aggccggcat
4621 tttcagcgtg acatcattct gtgggccgta cgctggtact gcaaatacgg catcagttac
4681 cgtgagctgc attttccgct gcataaccct gcttcggggt cattatagcg atttttttcgg
4741 tatatccatc ctttttcgca cgatatacag gattttgcca aagggttcgt gtagactttc
4801 cttggtgtat ccaacggcgt cagccgggca ggataggtga agtaggccca cccgcgagcg
4861 ggtgttcctt cttcactgtc ccttattcgc acctggcggt gctcaacggg aatcctgctc
4921 tgcgaggctg gcgggaactt cgaagttcct atactttcta gagaatagga acttcgaact
4981 gcaggtcgac ggatccCTGT TCGCGGTCGC CCTGTTCGCG TACCTGCTCG ACCTGGGCAG
5041 CAAGATGCTG GTGGTCGCCA AGCTGGAGCA CCACGAGCCG ATCGAGATCA TCGGCGACTG
5101 GCTGCGGTTC GCCGCGATCC GCAACGCGGG CGCGGCCTTC GGCTTCGGCG AGGCGTTCAC
5161 GATCATCTTC ACGGTGATCG CCGCTGCCGT GATCGTCGTG ATCGCCCGCC TCGCGCGCAA
5221 GCTGCACAGC CTGCCCTGGG CGATCGCGCT CGGCCTGCTG CTCGGCGGTG CCCTCGGCAA
5281 CCTCACCGAC CGGATCTTCC GTGCTCCCGG GGTCTTCGAG GGCGCGGTCG TGGACTTCAT
5341 CGCCCCCAAG CACTTCGCCG TCTTCAACCT CGCCGACTCG GCGATCGTGT GCGGCGGCAT
5401 CCTGATCGTG ATCCTCTCCT TCCGGGGCCT GGACCCGGAC GGGACCGTCC ACAAGGACTG
5461 ACCCCTCCGG TCCGGCTGTC CACAGGGACT GGTTGTC
//
```

Text S5. An example of the expected results following insertion of pJM016 into the M145 chromosome. A single crossover event would result in the disruption of the target gene by insertion of the entire plasmid. This was not the case in our example where instead a recombination event resulting in an unexpected result removing most of the vector and leaving a near perfect redirect deletion.
